# Performance of Intra-arrest Echocardiography: A Systematic Review

**DOI:** 10.5811/westjem.18440

**Published:** 2024-02-09

**Authors:** Yi-Ju Ho, Chih-Wei Sung, Yi-Chu Chen, Wan-Ching Lien, Wei-Tien Chang, Chien-Hua Huang

**Affiliations:** * National Taiwan University Hospital, Department of Emergency Medicine, Taipei, Taiwan; † National Taiwan University Hsin-Chu Hospital, Department of Emergency Medicine, Hsinchu, Taiwan; ‡ National Taiwan University, Institute of Epidemiology and Preventive Medicine, College of Public Health, Taipei, Taiwan; § National Taiwan University, College of Medicine, Department of Emergency Medicine, Taipei, Taiwan

**Keywords:** cardiac arrest, resuscitation, transthoracic echocardiography, transesophageal echocardiography

## Abstract

**Introduction:**

Intra-arrest transthoracic echocardiography (TTE) and transesophageal echocardiography (TEE) have been introduced in adult patients with cardiac arrest (CA). Whether the diagnostic performance of TTE or TEE is superior during resuscitation is unclear. We conducted a systematic review following PRISMA guidelines.

**Methods:**

We searched databases from PubMed, Embase, and Google Scholar and evaluated articles with intra-arrest TTE and TEE in adult patients with non-traumatic CA. Two authors independently screened and selected articles for inclusion; they then dual-extracted study characteristics and target conditions (pericardial effusion, aortic dissection, pulmonary embolism, myocardial infarction, hypovolemia, left ventricular dysfunction, and sonographic cardiac activity). We performed quality assessment using the Quality Assessment of Diagnostic Accuracy Studies Version 2 criteria.

**Results:**

A total of 27 studies were included: 14 studies with 2,145 patients assessed TTE; and 16 with 556 patients assessed TEE. A high risk of bias or applicability concerns in at least one domain was present in 20 studies (74%). Both TTE and TEE found positive findings in nearly one-half of the patients. The etiology of CA was identified in 13% (271/2,145), and intervention was performed in 38% (102/271) of patients in the TTE group. In patients who received TEE, the etiology was identified in 43% (239/556), and intervention was performed in 28% (68/239). In the TEE group, a higher incidence regarding the etiology of CA was observed, particularly for those with aortic dissection. However, the outcome of those with aortic dissection in the TEE group was poor.

**Conclusion:**

While TEE could identify more causes of CA than TTE, sonographic cardiac activity was reported much more in the TTE group. The impact of TTE and TEE on the return of spontaneous circulation and further survival was still inconclusive in the current dataset.

Population Health Research Capsule What do we already know about this issue?

*Transthoracic echocardiography (TTE) delays chest compressions; transesophageal echocardiography (TEE) offers real-time visualization without interrupting compressions.*

What was the research question?

*Is the diagnostic performance of TTE superior to TEE?*

What was the major finding of the study?

*The etiology of cardiac arrest (CA) was identified in 13% of patients through TTE and in 43% of patients through TEE.*

How does this improve population health?

*While TEE could identify more causes of CA sonographic cardiac activity, it was reported much more in the TTE group. The impact of TTE and TEE on further survival are inconclusive.*

## INTRODUCTION

Ultrasound (US) is considered a valuable diagnostic tool when there is a clinical suspicion for a specific reversible cause in patients with cardiac arrest (CA).^1^ The use of US during resuscitation has become more common because of its non-invasive and readily accessible characteristics.^2^ ^,^ ^3^

Transthoracic echocardiography (TTE) has been introduced in resuscitative scenarios in recent decades.^2^ ^,^ ^4^ ^,^ ^5^ However, previous studies have shown that TTE lengthens a single pause for more than 17 seconds,^6^ ^,^ ^7^ possibly delaying chest compressions. Also, devices such as mechanical chest compression systems or defibrillation pads would interfere with image acquisition. By contrast, transesophageal echocardiography (TEE) could overcome such limitations, allowing real-time visualization of the heart without interrupting chest compressions.^8^ However, the disadvantages of TEE include high cost, the need for advanced operator skills, and the potential for iatrogenic trauma due to its invasive nature. Whether the diagnostic performance of TTE or TEE is superior during resuscitation is unclear. We conducted a systematic review of intra-arrest TTE and TEE on target conditions including pericardial effusion, cardiac tamponade, aortic dissection, pulmonary embolism (PE), myocardial infarction (MI), hypovolemia, left ventricular (LV) dysfunction, and sonographic cardiac activity.

## METHODS

We performed a systematic review following the latest statement of the Preferred Reporting Items for Systematic Reviews and Meta-Analysis (PRISMA 2022). This review protocol was submitted to the International Prospective Register of Systematic Reviews (PROSPERO) on March 17, 2022 (registration number: CRD42022310670).

### Data Sources and Search Strategy

Two independent investigators searched literature published up to April 30, 2023, in PubMed, Embase, and Google Scholar without language or study-type restriction. Eligible trials were identified with the following keywords: “echocardiography, CA, resuscitation or rescue.”

### Study Selection

Two authors (YH and WL) independently examined references using titles and abstracts. Full texts of relevant studies were retrieved. The study selection criteria were framed using the PICOST (Population, Intervention, Comparator, Outcome, Study Design, Time frame) format as described in Table 1.

**Table 1.** Eligibility criteria for study selection.

	Inclusion criteria	Exclusion criteria
Population	Adult non-traumatic patients with out-of-hospital and in-hospital cardiac arrest	Traumatic arrest and pediatric population
Intervention	Intra-arrest TTE/TEE	Post-arrest TTE/TEE
Comparative	Standard resuscitation according to ALS guidelines	
Outcome	Identification of the target conditions	
Study type	Observational studies (prospective and retrospective) and interventional studies (randomized and non-randomized)	Case reports and case series, animal studies, review articles, guidelines, and editorials
Time	No limitations on the publication period	

*TEE*, transesophageal echocardiography; *TTE*, transthoracic echocardiography; *ALS*, Advanced Life Support.

### Data Extraction and Quality Assessment

The two authors (YH and WL) extracted data from eligible studies including authors, publication year, study design, case numbers, gender, age, application of TTE or TEE, sonographers, sonographic diagnoses, reference standard, and rate of return of spontaneous circulation (ROSC) or survival. Quality assessment was performed using the Quality Assessment of Diagnostic Accuracy Studies Version 2 (QUADAS-2) criteria, which is an adequate tool for diagnostic test accuracy in systematic reviews.^9^ The two authors independently evaluated each included study; any inconsistency or disagreement was resolved upon detailed discussion.

### Outcome Measures

The primary outcome was the incidence proportions of target conditions including pericardial effusion, cardiac tamponade, aortic dissection, PE, MI, hypovolemia, LV dysfunction, and sonographic cardiac activity by intra-arrest TTE/TEE.

### Data Synthesis, Statistical Analysis, and Sensitivity Analysis

Due to marked heterogeneity among the included studies, conducting a robust meta-analysis was not feasible. Thus, we used a narrative synthesis to present the study results. We calculated the pooled incidence proportions of target conditions of TTE and TEE and present them as proportion and 95% confidence intervals (CI) using Comprehensive Meta-Analysis V4.0 software (Biostat Inc, Englewood, NJ).

## RESULTS

### Literature Search and Study Selection Results

Our literature search identified a total of 333 results from PubMed, Embase, and Google Scholar. After duplicates were removed, we screened 308 titles for inclusion with 34 excluded for not meeting the study criteria. We also excluded 31 meta-analyses or systematic reviews and 61 case reports, case series, and animal studies. Of the 182 remaining studies that underwent a thorough full-text retrieval and review, we selected 27 for final review (Figure).^2^ ^,^ ^3^ ^,^ ^10^ ^–^ ^34^

**Figure.**
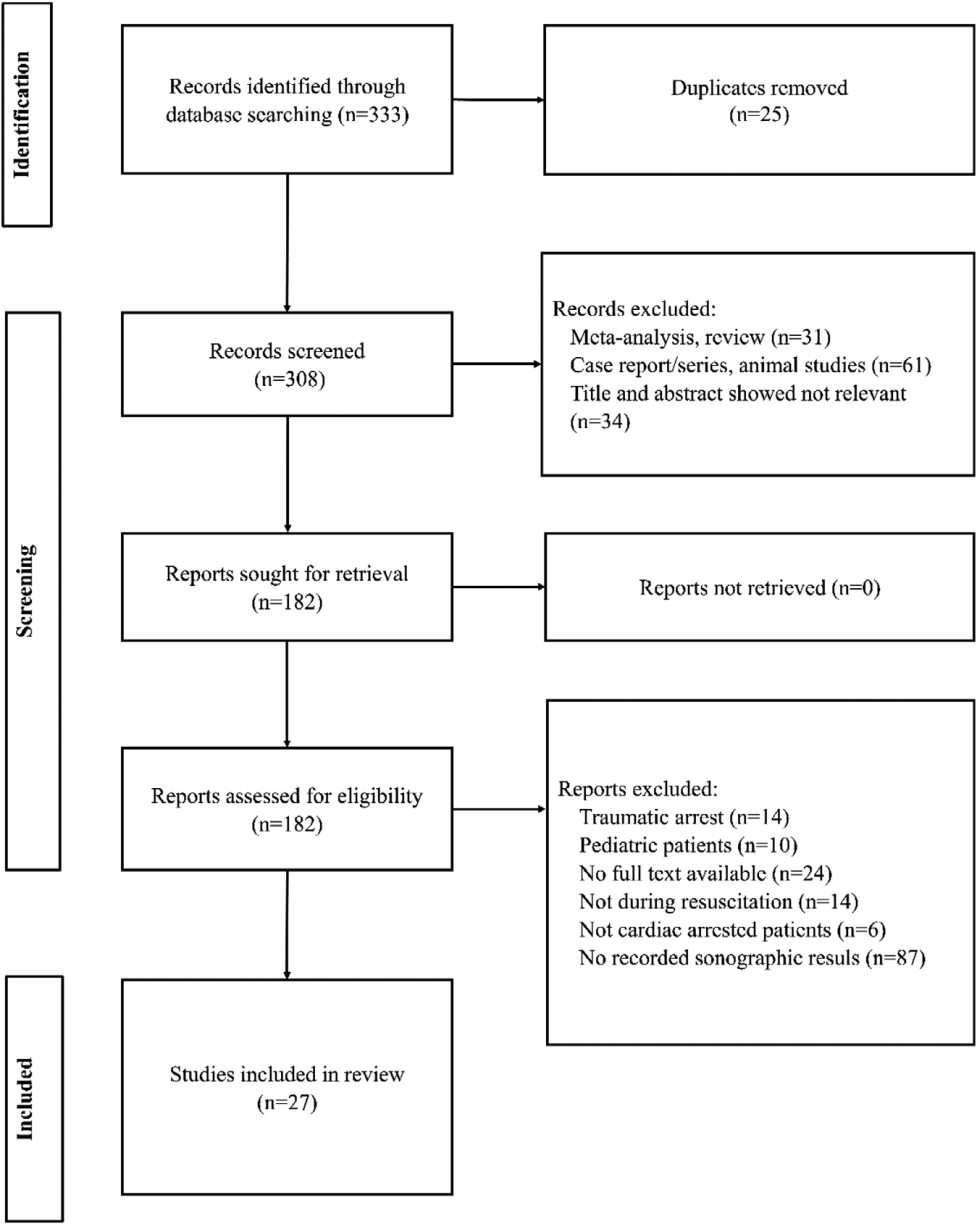
The Preferred Reporting Items for Systematic Reviews and Meta-Analyses (PRISMA) diagram.

### Summary of Studies

Eleven studies assessed TTE,^2^ ^,^ ^3^ ^,^ ^10^ ^–^ ^20^ 13 assessed TEE,^10^ ^,^ ^13^ ^,^ ^21^ ^–^ ^33^ and three included both (Supplementary Table 1).^10^ ^,^ ^13^ ^,^ ^34^ Of the total 2,701 patients included, 2,145 received TTE, and 556 received TEE. One patient was excluded due to a traumatic rupture of the thoracic aorta.^21^ Nine studies included patients with out-of-hospital CA (OHCA), seven included patients with in-hospital CA, and another 11 assessed a mix. Echocardiography was performed by emergency physicians in 17 studies.

### Risk of Bias and Concerns of Applicability

A high risk of bias or applicability concerns in at least one domain was present in 20 studies (74%) (Table 2). The risk of bias was unclear in 10 studies (38%) due to a lack of information regarding patient selection. The risk was high in 15 studies (58%) due to a convenience sample; only one study rated low risk enrolled a consecutive sample.^14^ In two studies in which TTE, TEE, or blood sampling analysis was performed at the discretion of physicians^19^ the risk of bias was rated high because of concern for the index test. The risk of bias was unclear in 23 studies (88%) related to a lack of standardized reference and information regarding the flow and timing. Low risks of bias related to reference standards and flow and timing were rated in the other four studies in which all images were reviewed (ie, uniform confirmatory testing) and inter-rater reliability was assessed.^26^ ^,^ ^31^ ^,^ ^32^ ^,^ ^34^

**Table 2.** The quality assessment of diagnostic accuracy studies (QUADAS)-2 risk of bias assessment of the included studies.

	Risk of bias				Concerns regarding applicability		
Study	Patient selection	Index test	Reference standard	Flow and timing	Patient selection	Index test	Reference standard
Transthoracic echocardiography							
Varriale et al (1997)	H	L	U	U	L	H	U
Kürkciyan et al (2000)	L	H	L	L	L	H	L
Tayal et al (2003)	U	L	U	U	H	L	U
Breitkreutz et al (2010)	H	L	U	U	H	L	U
Chardoli et al (2012)	H	L	U	U	H	L	U
Shillcutt et al (2012)	H	L	U	U	H	H	U
Flato et al (2015)	L	L	U	U	H	L	U
Gaspari et al (2016)	H	L	U	U	L	L	U
Zengin et al (2016)	U	L	U	U	L	L	U
Chua et al (2017)	U	L	U	U	L	L	U
Lien et al (2018)	H	L	U	U	L	L	U
Balderston et al (2021)	H	L	U	U	L	L	U
Heikkilä1 et al (2023)	U	H	U	U	L	H	U
Lien et al (2023)	H	L	U	U	L	L	U
Transesophageal echocardiography							
Varriale et al (1997)	H	L	U	U	L	H	U
van der Wouw et al (1997)	U	L	U	U	L	L	U
Comess et al (2000)	U	L	U	U	H	L	U
Kürkciyan et al (2000)	L	H	L	L	L	H	L
Miyake et al (2004)	H	L	U	U	H	L	U
Lin et al (2006)	U	L	U	U	L	L	U
Mentsoudis et al (2006)	U	L	U	U	L	L	U
Shillcutt et al (2012)	H	L	U	U	H	H	U
Hilberath et al (2014)	H	L	L	L	H	L	L
Burrage et al (2015)	U	L	U	U	H	L	U
Arntfield et al (2016)	U	L	U	U	U	L	U
Teran et al (2019)	H	L	U	U	L	L	U
Jung et al (2020)	U	L	U	U	L	L	U
Kim et al (2020)	H	L	L	L	L	L	L
Jung et al (2022)	H	L	L	L	L	L	L
Poppe (2023)	H	L	U	U	L	L	U

*H*, high risk of bias; *L*, low risk of bias; *U*, unclear risk of bias.

The risk of bias for applicability was high for patient selection in nine studies (35%) because of the enrollment of patients with hemodynamic instability or pulseless electrical activity.^2^ ^,^ ^11^ ^–^ ^14^ ^,^ ^22^ ^,^ ^26^ ^–^ ^28^ For applicability to the index test, four studies were evaluated with a high risk due to large variations in diagnostic assessment.^10^ ^,^ ^13^ ^,^ ^19^ ^,^ ^34^ Four studies in which reference standards were provided were rated with a low risk of applicability in reference standards.^26^ ^,^ ^31^ ^,^ ^32^ ^,^ ^34^ However, the remaining were unclear.

### Performance of TTE and TEE Among the Target Conditions

Transthoracic echocardiography and TEE found positive findings in 51% (1,101/2,145) and 47% (264/556) of patients, respectively. The most common finding was the presence of sonographic cardiac activity in 855 patients (830 in the TTE group and 25 in the TEE group). The etiology of CA was identified in 13% (271/2,145) of patients with TTE and 43% (239/556) of patients with TEE (Table 3, Supplementary Table 2). A high incidence proportion was observed in the target condition in the TEE group, particularly in those with aortic dissection. However, the outcome of patients with aortic dissection was poor.

**Table 3.** Pooled results of target findings on transthoracic echocardiography and transesophageal echocardiography in patients with cardiac arrest.

Findings	Transthoracic echocardiography N; incidence proportion [95% CI]	Transesophageal echocardiography N; incidence proportion [95% CI]
Pericardial effusion	113; 0.068 [0.046; 0.100]	35; 0.117 [0.056; 0.226]
Cardiac tamponade	30; 0.059 [0.041; 0.083]	25; 0.095 [0.036; 0.228]
Aortic dissection	10; 0.023 [0.008; 0.064]	54; 0.119 [0.074; 0.186]
Pulmonary embolism	34; 0.053 [0.021; 0.126]	70; 0.220 [0.116; 0.378]
Myocardial infarction	4; 0.022 [0.002; 0.192]	28; 0.291 [0.131; 0.528]
Hypovolemia	26; 0.044 [0.013; 0.142]	6; 0.147 [0.067; 0.291]
LV dysfunction	84; 0.181 [0.086; 0.343]	46; 0.535 [0.170; 0.866]
Sonographic cardiac activity	830; 0.488 [0.374; 0.604]	25; 0.243 [0.138; 0.390]

*LV*, left ventricular; *CI*, confidence interval.

The summary of detailed sonographic findings is listed in Supplementary Table 3.

### Intervention According to Sonographic Findings

Excluding patients with sonographic cardiac activity, the effect on management was observed in 38% (102/271) of patients receiving TTE and 28% (68/239) of those receiving TEE. The most common intervention was pericardial effusion drainage, which was performed in 51% (58/113) of patients in the TTE group, and 51% (18/35) in the TEE group. Surgery was performed on one patient (1/10, 10%) with suspected aortic dissection in the TTE group^17^ and two (2/54, 4%) in the TEE group.^2^ Thrombolysis/embolectomy was performed in seven patients (7/34, 21%) and 28 patients (28/70, 40%) with suspected PE in the TTE^3^ ^,^ ^16^ and TEE^10^ ^,^ ^24^ ^,^ ^25^ ^,^ ^27^ ^,^ ^30^ ^,^ ^31^ groups, respectively. Coronary angiography or bypass was performed on one patient (1/4, 25%) with suspected MI by TTE^17^ and 11 patients (11/28, 39%) by TEE.^21^ ^,^ ^24^ ^,^ ^25^ Administration of fluid was reported in 13 patients (13/26, 50%) with hypovolemia by TTE,^2^ ^,^ ^12^ and four patients (4/6, 67%) by TEE.^13^ ^,^ ^24^ ^,^ ^25^ Inotropic therapy was administered in 26% (22/84) of patients with LV dysfunction by TTE^2^ and 11% (5/46) by TEE.^25^ ^,^ ^27^

## DISCUSSION

Intra-arrest TTE was performed in 2,145 patients in 14 studies, and TEE was used in 556 patients in 16 studies. Both TTE and TEE found positive findings in nearly one-half of the patients. The etiology of CA was identified in 13% of patients with TTE and 43% of patients with TEE. Prompt therapy was administered in 38% of patients with TTE-positive findings and 28% of those with TEE-positive findings. In the TEE group, a higher incidence proportion was observed in identifying the etiology of CA, particularly for those with aortic dissection. However, a high degree of heterogeneity in reference standards and small-sample size precluded further meta-analysis for the diagnostic performance of intra-arrest TTE and TEE.

The major weakness of the included studies is that the reference standards are inconsistent. Applying a uniform standard to all target conditions is not easy, and image review may be an effective solution.^26^ ^,^ ^31^ ^,^ ^32^ ^,^ ^34^ The autopsy can be regarded as the gold standard and was performed in three studies,^21^ ^,^ ^22^ ^,^ ^25^ but the reference standards are diverse in patients with ROSC. Using specific management as reference standards to judge target conditions is not feasible. For example, patients with suspected PE do not receive pericardiocentesis. Also, even failure in pericardiocentesis does not indicate the absence of pericardial effusion. By contrast, pericardial effusion drainage is rarely performed in a small amount of effusion. True-positive cases were often reported, but verification bias existed. Information was limited in the true-negative, false-positive, or false-negative cases. Therefore, this systematic review presented the incidence of target conditions and could not further explore whether TTE or TEE was better during resuscitation.

One of the most important indicators of the likelihood of ROSC or survival is the presence of sonographic cardiac activity.^35^ Sonographic cardiac activity was much more frequently detected by TTE than by TEE (830 vs 25) in our review. Interestingly, regional wall motion abnormality suggestive of MI and LV dysfunction was detected in the presence of sonographic cardiac activity. Even adding the numbers of these conditions, the total number was still higher in patients with TTE. Whether it occurred due to taking a longer time to set up TEE (possibly resulting in resuscitation time bias^36^) was uncertain. Further studies are needed to determine whether TEE can better characterize intra-arrest myocardial movement or cardiac activity detected by TEE is under-reported. The impact of TTE and TEE on ROSC and further survival was still inconclusive in the current dataset.

The most common intervention was pericardial effusion drainage during resuscitation. The outcomes varied depending on the etiology of effusions. One patient with cardiac tamponade and LV free wall rupture secondary to transmural MI by TTE died after an exploratory thoracotomy.^14^ Return of spontaneous circulation was not achieved in one patient with tamponade and aortic dissection by TTE.^17^ One patient had cardiac tamponade that was not visible on TTE owing to poor acoustic windows but was evident on TEE and survived after surgical treatment.^21^ One patient with tamponade and right ventricular rupture survived to discharge after receiving an emergent wall repair.^25^ Early termination of resuscitation was conducted in six patients with tamponade due to myocardial rupture and one patient with tamponade due to aortic dissection by TEE.^21^ ^,^ ^31^

The incidence of aortic dissection was higher by TEE, which was related to direct visualization of the aortic root and descending aorta by the long-axis and short-axis views. However, the data should be cautiously interpreted because Jung et al reported 19 patients ^30^ and Kim et al reported 10 patients,^31^ which could skew the results. Moreover, the outcomes were poor in that only one patient with TTE^20^ and two with TEE^23^ had ROSC.

Pulmonary embolism is the most reported finding by TEE, which illustrates the thrombi directly^13^ ^,^ ^22^ ^,^ ^24^ ^,^ ^25^ ^,^ ^27^ ^,^ ^30^ or obstruction to color flow in the pulmonary artery.^10^ Transthoracic echocardiography uses the indirect sign of right ventricular dilatation, indicative of PE.^2^ ^,^ ^3^ ^,^ ^14^ ^–^ ^16^ However, false-positive and false-negative cases were reported, and not all the patients received thrombolysis or thrombolectomy. Van de Wouw et al reported one had a TEE diagnosis of PE but no embolus was found at autopsy.^21^ Comess et al reported bilateral peripheral pulmonary emboli at autopsy but not seen by TEE.^22^ Jung et al reported one patient with initial negative TEE findings had thrombi in the main pulmonary artery at the final review, and one patient with saline bubbles in the pulmonary artery was misinterpreted as PE.^30^

Gaspari et al reported one of the 15 patients with suspected PE receiving thrombolysis survived hospital discharge.^3^ Chua et al reported one of four patients suggestive of massive PE by TTE receiving thrombolysis survived to discharge.^16^ Although the ROSC rates of PE by TTE or TEE were still lower, they were better than those of aortic dissection.

The sonographic finding suggestive of MI on intra-arrest TTE and TEE is regional wall motion abnormality in the presence of sonographic cardiac activity. Van de Wouw et al reported two patients had MI at autopsy that could not be demonstrated with TEE owing to lack of spontaneous rhythm.^21^ Lien et al reported extensive anterior wall akinesia of the left ventricle that was identified in one patient with pulseless electrical activity.^17^

A low LV end-diastolic volume is a characteristic finding indicative of hypovolemia by TTE^2^ and TEE.^13^ ^,^ ^24^ Fluid resuscitation was reported in two studies of TTE,^2^ ^,^ ^12^ and three studies of TEE.^13^ ^,^ ^24^ ^,^ ^25^ Lactated Ringer solution infusion in OHCA increased the likelihood of prehospital ROSC^37^; however, the information regarding the details of fluid was lacking in the included studies.

Reduced LV function presented as a common finding by intra-arrest TTE and TEE. Instead of precise measurement, reduced LV function is estimated by visual assessment (eyeballing) via ECHO of an ejection fraction less than 45–55%.^13^ ^,^ ^14^ ^,^ ^18^ Also, the ventricular function could be assessed only during intervals of spontaneous cardiac contraction.^22^ Inotropic therapy was administered in one-fourth of patients with LV dysfunction by TTE^2^ and one-tenth of those by TEE.^25^ ^,^ ^27^

Intra-arrest TTE is a convenient imaging modality, but it is challenged by the technical difficulty in obtaining adequate cardiac windows during the pauses and potential delays in chest compressions.^7^ Transesophageal echocardiography has been recognized as an alternative without interfering with ongoing resuscitation efforts. It provides real-time feedback on the location of chest compressions and the quality of cardiopulmonary resuscitation (CPR).^38^ However, TEE has disadvantages such as high cost, high level of operator skill training required, and potentially iatrogenic trauma including oropharyngeal esophageal and gastric lacerations, and perforation.^39^ Three studies reported no complications or delays in resuscitation procedures.^22^ ^,^ ^32^ ^,^ ^33^ The transgastric view was excluded from the TEE protocol to avoid potential complications or to lessen interference with the chest compression procedure.^30^ ^,^ ^31^

## LIMITATIONS

There were limitations in this review. First, the selected studies were highly heterogeneous, small-sized samples, with a lack of reference groups and standardized confirmation tests. Most of the patients were collected from a convenience sample from a single institution. Beyond the selection bias, resuscitation facilities and interventions for specific diseases may differ depending on the institution’s capability, limiting the generalizability. Future studies assessing the diagnostic accuracy of US in patients with CA should avoid methodological flaws; a randomized controlled trial comparing TTE with TEE would be a solution. Second, the detailed training background of sonographers was unclear in some of the included studies.^10^ ^,^ ^13^ ^,^ ^19^ ^,^ ^21^ ^,^ ^23^ ^–^ ^25^ ^,^ ^34^ The 2022 guidelines suggest US can be performed by experienced personnel without interrupting CPR.^1^ Third, the timing of the introduction of TTE and TEE was not clear in the studies; so resuscitation time bias could not be estimated.^39^ Lien et al used US within 10 minutes of Advanced Life Support (ALS), and Gaspari et al introduced US after five minutes of ALS.^3^ ^,^ ^17^ On the other hand, Jung et al and Kim et al introduced TEE after 10 minutes of ALS.^30^ ^,^ ^31^ Lien et al reported that TTE was performed in patients with longer resuscitation time.^20^

Lastly, the impact of TTE and TEE on ROSC and further survival was not thoroughly discussed. The etiology of CA was identified in 13% of patients with TTE and 43% of patients with TEE. Among them, approximately 20–25% of patients with each target condition achieved ROSC except those with aortic dissection (10%). However, the resuscitation data associated with ROSC such as witnessed arrest, early ALS, and early defibrillation were not presented. Also, patients with early ROSC before US was attempted were excluded from some studies.^3^ ^,^ ^30^ Future research would focus on evaluating the values of TTE and TEE on ROSC, hospital survival, or long-term neurological outcomes.

## CONCLUSION

Transesophageal echocardiography could identify more causes of cardiac arrest than transthoracic echocardiography. However, sonographic cardiac activity, indicative of better rates of return of spontaneous circulation, was reported much more in the TTE group. The impact of TTE and TEE on ROSC and further survival was inconclusive in the current data. A high degree of heterogeneity in patient selection and a lack of reference standards precluded further meta-analysis for the diagnostic performance of intra-arrest TTE and TEE.

## Supplementary Information




