# Supplementary material for: Performance of Intra-arrest Echocardiography: A Systematic Review
Source: West J Emerg Med. 2024 Feb 9;25(2):166–74. doi: 10.5811/westjem.18440 (PMC11000560; doi:10.5811/westjem.18440)
Supplement: Supplementary file 1 [file wjem-25-166-s001.docx]

**Supplementary Table 1.** Details of the included studies.

| Echocardiography | Study | Population | Arrest site | Sonographer |
| --- | --- | --- | --- | --- |
| TTE | Varriale 1997 (n=16) | IHCA | ED, ICU, OR, medical floors | Cardiologist |
|  | Kürkciyan 2000 (n=6) | OHCA, IHCA | ED | Emergency physician |
|  | Tayal 2003 (n=20) | OHCA, IHCA | ED | Emergency physician |
|  | Breitkreutz 2010 (n=100) | OHCA | EMS | Emergency physician |
|  | Chardoli 2012 (n=50) | OHCA, IHCA | ED | Emergency physician |
|  | Shillcut 2012 (n=4) | IHCA | OR, post-anesthesia care unit | Anesthesiologist |
|  | Flato 2015 (n=49) | IHCA | ICU | Intensivists |
|  | Zengin 2016 (n=179) | OHCA, IHCA | ED | Emergency physician |
|  | Gaspari 2016 (n=793) | OHCA | ED | Emergency physician |
|  | Chua 2017 (n=104) | OHCA | ED | Emergency physician |
|  | Lien 2018 (n=177) | OHCA | ED | Emergency physician |
|  | Balderston 2021 (n=126) | OHCA, IHCA | ED | Emergency physician |
|  | Heikkila 2023 (n=331) | OHCA | EMS | Emergency physician |
|  | Lien 2023 (n=190) | OHCA | ED | Emergency physician |
| TEE | Varriale 1997 (n=4) | IHCA | OR | Cardiologist |
|  | van der Wouw 1997 (n=47) | OHCA, IHCA | ED | not mentioned |
|  | Comess 2000 (n=35) | OHCA, IHCA | ED, ICU, ward | Cardiologist |
|  | Kürkciyan 2000 (n=18) | OHCA, IHCA | ED | Emergency physician |
|  | Miyaki 2004 (n=4) | OHCA, IHCA | ED, ICU | Cardiologist |
|  | Lin 2006 (n=10) | IHCA | OR | Anesthesiologist |
|  | Memtsoudis 2006 (n=22) | IHCA | OR | Cardiac anesthesiologist |
|  | Shillcut 2012 (n=4) | IHCA | OR, post-anesthesia care unit | Anesthesiologist |
|  | Hilberath 2014 (n=5) | IHCA | OR | Cardiac anesthesiologist |
|  | Burrage 2015 (n=6) | IHCA | OR | Cardiac anesthesiologist |
|  | Arntfield 2016 (n=23) | OHCA, IHCA | ED | Emergency physician |
|  | Teran 2019 (n=21) | OHCA | ED | Emergency physician |
|  | Jung 2020 (n=158) | OHCA | ED | Emergency physician |
|  | Kim 2021 (n=45) | OHCA, IHCA | ED | Emergency physician |
|  | Jung 2022 (n=97) | OHCA | ED | Emergency physician |
|  | Poppe 2023 (n=57) | OHCA, IHCA | ED | Emergency physician |

*TEE*, transesophageal echocardiography; *TTE*, transthoracic echocardiography; *OHCA*, out-of-hospital cardiac arrest; *IHCA*, in-hospital cardiac arrest; *ED*, emergency department; *ICU*, intensive care unit; *OR*, operating room; *EMS*, emergency medical services.

**Supplementary Table 2**. Incidence proportion of the target conditions.

| Target condition | Study | Incidence (%) | TP | FP | FN | TN | ROSC |
| --- | --- | --- | --- | --- | --- | --- | --- |
| Pericardial effusion | |  |  |  |  |  |  |
| TTE | Varriale 1997 (n=16) | 2/16 (13%) |  |  |  |  | 0 |
|  | Tayal 2003 (n=20) | 8/20 (40%) |  |  |  |  | 7/8 (88%) |
|  | Breitkreutz 2010 (n=100) | 5/100 (5%) |  |  |  |  | 3/5 (60%) |
|  | Chardoli 2012 (n=50) | 7/50 (14%) |  |  |  |  | 2/7 (29%) |
|  | Flato 2015 (n=49) | 2/49 (4%) |  |  |  |  | 1/2 (50%) |
|  | Zengin 2016 (n=179) | 10/179 (6%) |  |  |  |  | Survival to leaving ED, 5/10 (50%) |
|  | Gaspari 2016 (n=793) | 34/793 (4%) |  |  |  |  | Survival to discharge, 2/13 (15%) |
|  | Chua 2017 (n=104) | 4/104 (4%) |  |  |  |  |  |
|  | Lien 2018 (n=177) | 9/177 (5%) |  |  |  |  | 3/9 (33%) |
|  | Balderston 2021 (n=126) | 10/126 (8%) |  |  |  |  | - |
|  | Heikkila 2023 (n=331) | 17/331 (5%) |  |  |  |  | - |
|  | Lien 2023 (n=190) | 5/190 (3%) |  |  |  |  | 1/5 (20%) |
| TEE | van der Wouw 1997 (n=47) | 8/47 (17%) |  |  |  |  | Survival to discharge, 1/8 (13%) |
|  | Miyaki 2004 (n=4) | 2/4 (50%) |  |  |  |  | 0 |
|  | Memtsoudis 2006 (n=22) | 2/22 (9%) |  |  |  |  | 1/2 (50%) |
|  | Hilberath 2014 (n=5) | 5/5 (100%) | 5 | 0 | 0 | 0 | 5/5 (100%) |
|  | Burrage 2015 (n=6) | 1/6 (13%) |  |  |  |  | 0 |
|  | Jung 2020 (n=158) | 5/158 (3%) |  |  |  |  | 3/4 (75%) |
|  | Kim 2021 (n=45) | 3/45 (7%) | 3 | 0 | 0 | 42 | 0 |
|  | Jung 2022 (n=97)* | 3/97 (3%) |  |  |  |  | - |
|  | Poppe 2023 (n=57) | 6/57 (11%) |  |  |  |  | - |
|  | |  |  |  |  |  |  |
| Cardiac tamponade | |  |  |  |  |  |  |
| TTE | Varriale 1997 (n=16) | 2/16 (13%) |  |  |  |  | 0 |
|  | Tayal 2003 (n=20) | 3/20 (15%) |  |  |  |  | - |
|  | Breitkreutz 2010 (n=100) | 5/100 (5%) |  |  |  |  | 3/5 (60%) |
|  | Flato 2015 (n=49) | 2/49 (4%) |  |  |  |  | 1/2 (50%) |
|  | Zengin 2016 (n=179) | 10/179 (6%) |  |  |  |  | Survival to leaving ED, 5/10 (50%) |
|  | Lien 2018 (n=177) | 8/177 (5%) |  |  |  |  | 2/8 (25%) |
| TEE | van der Wouw 1997 (n=47) | 8/47 (17%) |  |  |  |  | Survival to discharge, 1/8 (13%) |
|  | Memtsoudis 2006 (n=22) | 2/22 (9%) |  |  |  |  | 1/2 (50%) |
|  | Hilberath 2014 (n=5) | 5/5 (100%) | 5 | 0 | 0 | 0 | 5/5 (100%) |
|  | Burrage 2015 (n=6) | 1/6 (13%) |  |  |  |  | 0 |
|  | Jung 2020 (n=158) | 4/158 (3%) |  |  |  |  | 3/4 (75%) |
|  | Kim 2021 (n=45) | 2/45 (4%) | 2 | 0 | 0 | 43 | 0 |
|  | Jung 2022 (n=97)* | 3/97 (3%) |  |  |  |  | - |
|  |  |  |  |  |  |  |  |
| Aortic dissection |  |  |  |  |  |  |  |
| TTE | Tayal 2003 (n=20) | 2/20 (10%) |  |  |  |  | - |
|  | Zengin 2016 (n=179) | 2/179 (1%) |  |  |  |  | Survival to discharge, 0 |
|  | Lien 2018 (n=177) | 1/177 (1%) |  |  |  |  | 0 |
|  | Lien 2023 (n=190) | 5/190 (3%) |  |  |  |  | 1/5 (20%) |
| TEE | van der Wouw 1997 (n=47) | 4/47 (9%) |  |  |  |  | Survival to discharge, 0 |
|  | Comess 2000 (n=35) | 2/35 (6%) |  |  |  |  | 0 |
|  | Miyaki 2004 (n=4) | 4/4 (100%) |  |  |  |  | 0 |
|  | Arntfield 2016 (n=23) | 2/23 (9%) |  |  |  |  | - |
|  | Jung 2020 (n=158) | 19/158 (12%) |  |  |  |  | 5/19 (26%) |
|  | Kim 2021 (n=45) | 10/45 (22%) | 10 | 0 | 0 | 35 | 1/10 (10%) |
|  | Jung 2022 (n=97)* | 6/97 (6%) |  |  |  |  | - |
|  | Poppe 2023 (n=57) | 7/57 (12%) |  |  |  |  | - |
|  | |  |  |  |  |  |  |
| Pulmonary embolism | |  |  |  |  |  |  |
| TTE | Kürkciyan 2000 (n=6) | 4/6 (67%) |  |  |  |  | - |
|  | Breitkreutz 2010 (n=100) | 4/100 (4%) |  |  |  |  | 2/4 (50%) |
|  | Flato 2015 (n=49) | 3/49 (6%) |  |  |  |  | - |
|  | Gaspari 2016 (n=793) | 15/793 (2%) |  |  |  |  | Survival to discharge, 1/15 (7%) |
|  | Zengin 2016 (n=179) | 4/179 (2%) |  |  |  |  | 3/4 (75%) |
|  | Chua 2017 (n=104) | 4/104 (4%) |  |  |  |  | 1/4 (25%) |
| TEE | van der Wouw 1997 (n=47) | 6/47 (13%) |  |  |  |  | Survival to discharge, 0 |
|  | Varriale 1997 (n=4) | 1/4 (25%) |  |  |  |  | Survival to discharge, 1/1 (100%) |
|  | Comess 2000 (n=35) | 8/35 (23%) |  |  |  |  | 2/9 (22%) |
|  | Kürkciyan 2000 (n=18) | 12/18 (67%) | 12 | 0 | 6 | 0 | - |
|  | Lin 2006 (n=10) | 2/10 (20%) |  |  |  |  | 2/2 (100%) |
|  | Memtsoudis 2006 (n=22) | 8/22 (36%) |  |  |  |  | 5/8 (63%) |
|  | Shillcut 2012 (n=4) | 2/4 (50%) |  |  |  |  | Survival to discharge, 1/2 (50%) |
|  | Burrage 2015 (n=6) | 1/6 (13%) |  |  |  |  | 1/1 (100%) |
|  | Teran 2019 (n=21) | 12/21 (54%) |  |  |  |  | - |
|  | Jung 2020 (n=158) | 8/158 (5%) |  |  |  |  | 5/8 (63%) |
|  | Kim 2021 (n=45) | 5/45 (11%) | 5 | 0 | 0 | 40 | 0 |
|  | Jung 2022 (n=97)* | 5/97 (5%) |  |  |  |  | - |
|  | |  |  |  |  |  |  |
| Myocardial infarction | |  |  |  |  |  |  |
| TTE | Flato 2015 (n=49) | 3/49 (6%) |  |  |  |  | - |
|  | Lien 2018 (n=177) | 1/177 (1%) |  |  |  |  | 1/1 (100%) |
| TEE | van der Wouw 1997 (n=47) | 21/47 (45%) |  |  |  |  | Survival to discharge, 2/21 (10%) |
|  | Comess 2000 (n=35) | 2/35 (6%) |  |  |  |  | 0 |
|  | Lin 2006 (n=10) | 5/10 (50%) |  |  |  |  | 4/5 (80%) |
|  | Memtsoudis 2006 (n=22) | 6/22 (27%) |  |  |  |  | 6/6 (100%) |
|  |  |  |  |  |  |  |  |
| Hypovolemia |  |  |  |  |  |  |  |
| TTE | Breitkreutz 2010 (n=100) | 2/100 (2%) |  |  |  |  | - |
|  | Zengin 2016 (n=179) | 4/179 (2%) |  |  |  |  | Survival to leaving ED, 2/4 (50%) |
|  | Chardoli 2012 (n=50) | 11/50 (22%) |  |  |  |  | 4/11 (36%) |
|  | Flato 2015 (n=49) | 4/49 (8%) |  |  |  |  | - |
|  | Heikkila 2023 (n=331) | 5/331 (2%) |  |  |  |  | - |
| TEE | Lin 2006 (n=10) | 1/10 (10%) |  |  |  |  | 0 |
|  | Memtsoudis 2006 (n=22) | 3/22 (14%) |  |  |  |  | 2/3 (67%) |
|  | Shillcut 2012 (n=4) | 1/4 (25%) |  |  |  |  | Survival to discharge, 1/1 (100%) |
|  | Burrage 2015 (n=6) | 1/6 (13%) |  |  |  |  | 0 |
|  | |  |  |  |  |  |  |
| Left ventricular dysfunction | |  |  |  |  |  |  |
| TTE | Breitkreutz 2010 (n=100) | 22/100 (22%) |  |  |  |  | 11/22 (50%) |
|  | Shillcut 2012 (n=4) | 1/4 (25%) |  |  |  |  | Survival to discharge, 1/1 (100%) |
|  | Flato 2015 (n=49) | 11/49 (22%) |  |  |  |  | - |
|  | Zengin 2016 (n=179) | 7/179 (4%) |  |  |  |  | Survival to leaving ED, 3/7 (43%) |
|  | Balderston 2021 (n=126) | 43/126 (43%) |  |  |  |  | - |
| TEE | Comess 2000 (n=35) | 20/35 (57%) |  |  |  |  | 4/20 (20%) |
|  | Memtsoudis 2006 (n=22) | 2/22 (9%) |  |  |  |  | 1/2 (50%) |
|  | Burrage 2015 (n=6) | 3/6 (50%) |  |  |  |  | 2/3 (67%) |
|  | Teran 2019 (n=21) | 21/21 (100%) |  |  |  |  | - |
|  |  |  |  |  |  |  |  |
| Cardiac activity |  |  |  |  |  |  |  |
| TTE | Tayal 2003 (n=20) | 12/20 (60%) |  |  |  |  | 8/12 (67%) |
|  | Breitkreutz 2010 (n=100) | 38/100 (38%) |  |  |  |  | 21/38 (55%) |
|  | Chardoli 2012 (n=50) | 39/50 (78%) |  |  |  |  | 17/39 (44%) |
|  | Flato 2015 (n=49) | 27/49 (55%) |  |  |  |  | 19/27 (70%) |
|  | Zengin 2016 (n=179) | 104/179 (58%) |  |  |  |  | 55/104 (53%) |
|  | Gaspari 2016 (n=793) | 263/793 (33%) |  |  |  |  | 134/263 (51%) |
|  | Chua 2017 (n=104) | 26/104 (25%) |  |  |  |  | 13/26 (50%) |
|  | Lien 2018 (n=177) | 47/177 (27%) |  |  |  |  | 45/47 (96%) |
|  | Balderston 2021 (n=126) | 121/126 (96%) |  |  |  |  | - |
|  | Heikkila 2023 (n=331) | 68/331 (21%) |  |  |  |  | - |
|  | Lien 2023 (n=190) | 85/190 (45%) |  |  |  |  | 64/85 (75%) |
| TEE | Varriale 1997 (n=4) | 2/4 (50%) |  |  |  |  | 2/2 (100%) |
|  | Teran 2019 (n=21) | 5/21 (24%) |  |  |  |  | - |
|  | Poppe 2023 (n=57) | 18/57 (32%) |  |  |  |  | 30-day survival, 3/18 (17%) |

*TEE*, transesophageal echocardiography; *TTE*, transthoracic echocardiography; *ED*, emergency department; *TP*, true positive; *FP*, false positive; *FN*, false negative; *TN*, true negative; *ROSC*, return of spontaneous circulation.

*Jung et al used image review as the reference standard; however, the true-positive cases of the target conditions could not be retrieved due to the disconcordance between the initial and final reviews.

| **Supplemental Table 3.** Sonographic findings of transthoracic echocardiography and transesophageal echocardiography. | | |
| --- | --- | --- |
|  | Transthoracic echocardiography | Transesophageal echocardiography |
| Pericardial effusion | The anechoic area within the pericardium.  The amount could be estimated by the thickness.  Small < 10 mm (<100 ml)  Moderate 10-20mm (100-500 ml)  Severe > 20mm (>500 ml) | The anechoic area within the pericardium. The amount could be estimated by the thickness.  Small < 10 mm (<100 ml)  Moderate 10-20 mm (100-500 ml)  Severe > 20mm (>500 ml) |
|  |  |  |
| Cardiac tamponade | The presence of pericardial effusion with  the following sign.  Early sign: RA systolic collapsed.  Late sign: RV diastolic collapsed. | The presence of pericardial effusion with the following sign.  Early sign: RA systolic collapsed.  Late sign: RV diastolic collapsed. |
|  |  |  |
| Aortic dissection | Intimal flap in the ascending aorta. | Intimal flap in the ascending/aortic arch/descending aorta at the ME-SAX and upper esophageal level. |
| Pulmonary embolism | Indirect signs due to increased RV pressure, including  RV enlargement, D-shaped LV, or McConnell sign. | Direct signs include visible thrombus at the pulmonary artery or right chamber.  Indirect signs include RV enlargement, D-shaped LV, or McConnell sign. |
|  |  |  |
| Myocardial infarction | RWMA at the parasternal short-axis view. | RWMA at the ME-SAX view or the transgastric view. |
| Hypovolemia | IVC diameter <1.5cm or collapsibility index>50%  indicate hypovolemia | Kissing of the papilla muscles of LV at the transgastric view. |
| Sonographic cardiac activity | Visualized cardiac movement. | Visualized cardiac movement. |
| *RA*, right atrium; *RV*, right ventricle; *LV*, left ventricle; *RWMA*, regional wall motion abnormality; *IVC*, inferior vena cava; *ME-SAX*, midesophageal short-axis. | | |
